# A Qualitative Exploration of Sources of Help for Mental Illness in Arabic-, Mandarin-, and Swahili-Speaking Communities in Sydney, Australia

**DOI:** 10.3390/ijerph20105862

**Published:** 2023-05-18

**Authors:** Klimentina Krstanoska-Blazeska, Andre Renzaho, Ilse Blignault, Bingqin Li, Nicola Reavley, Shameran Slewa-Younan

**Affiliations:** 1 Translational Health Research Institute, School of Medicine, Western Sydney University, Campbelltown 2571, Australia; 2 Social Policy Research Centre, University of New South Wales, Sydney 2052, Australia; 3 Centre for Mental Health, Melbourne School of Population and Global Health, University of Melbourne, Melbourne 3010, Australia; 4 School of Medicine, Western Sydney University, Campbelltown 2571, Australia

**Keywords:** culturally and linguistically diverse, mental illness, sources of help, Arabic-speaking, Congolese, Mandarin-speaking, Swahili-speaking, qualitative research, Australia

## Abstract

Despite being disproportionately affected by poor mental health, culturally and linguistically diverse (CaLD) individuals seek help from mental health services at lower rates than others in the Australian population. The preferred sources of help for mental illness amongst CaLD individuals remain poorly understood. The aim of this study was to explore sources of help in Arabic-, Mandarin-, and Swahili-speaking communities in Sydney, Australia. Eight focus-group discussions (n = 51) and twenty-six key informant interviews were undertaken online using Zoom. Two major themes were identified: informal sources of help and formal sources of help. Under the informal sources of help theme, three sub-themes were identified: social, religious, and self-help sources. All three communities strongly recognised the role of social sources of help, with more nuanced roles held by religion and self-help activities. Formal sources of help were described by all communities, although to a lesser extent than informal sources. Our findings suggest that interventions to support help-seeking for all three communities should involve building the capacity of informal sources of help, utilising culturally appropriate environments, and the collaboration between informal and formal sources of help. We also discuss differences between the three communities and offer service providers insights into unique issues that require attention when working with these groups.

## 1. Introduction

Australia’s cultural landscape is richly diverse, comprising a mixture of languages, ethnicities, traditions, values, and beliefs. Just over half (51.5%) of Australians report having been born overseas (first-generation immigrants) or having a parent born overseas [1]. Two of the most spoken languages in the home other than English in Australia are Mandarin (2.7%) followed by Arabic [1.4%; 1]. The majority of migrants enter through the family and skills migration stream [2]. Additionally, Australia’s Refugee and Humanitarian Program offers durable solutions to individuals forced to flee their home countries due to persecution or war [3]. Irrespective of arrival pathway, all migrants face resettlement challenges, such as loss of support structures, language barriers, discrimination, and acculturative stress, which can contribute to poor mental health [4,5,6,7]. This, along with the exposure to traumatic events pre-migration for refugees and asylum seekers, places them at a further risk of developing post-traumatic stress disorder and depression [8,9,10].

Effective patterns of service utilisation for mental illness among culturally and linguistically diverse (CaLD) individuals are difficult to ascertain given the lack of mental illness prevalence data in these groups [11]. The existing evidence base indicates that CaLD individuals may be accessing mental health services at reduced rates compared to those from a non-CaLD background [12,13]. McDonald and Steel [14] identified that Arabic-speaking and Chinese immigrants were less likely to utilise specialist psychiatric services and had lower voluntary hospitalisation rates compared to the general population of New South Wales. This reduced engagement and delayed seeking help from professional mental health service providers can result in CaLD individuals then presenting to mental health services in crisis [15], possibly accounting for the finding of higher rates of involuntary hospitalisations [14].

### 1.1. Sources of Help

Arabic-speaking individuals, as well as those of Chinese and African backgrounds have been shown to prefer to seek informal sources of help, such as from family and friends [9,16,17,18]. One study found that Asian-born patients were less likely to have contact with allied health professionals than Australian-born patients [19]. Arabic-speaking individuals, as well as those from an African background also commonly utilise religious strategies, such as prayer, to manage mental illness [20,21,22], and will often seek help from a religious leader as the first point of contact [21]. Chinese individuals residing in New South Wales also endorsed seeking help from a church/temple or religious/faith-based organisation after seeking help from friends and family [23]. Such endorsements may represent a lack of familiarity with or availability of mental health services in their country of origin [24,25,26]. Migrants may be more likely to be affiliated with religious and community leaders due to acculturative stress and separation from traditional support structures in their home countries [21,27].

### 1.2. Barriers to Help Seeking

Mental-health-related stigma has been shown to be a considerable barrier to seeking professional help in the three communities [18,21,28,29]. Other barriers to professional help seeking include confidentiality concerns due to the need for interpreters; experiences of racism and discrimination; low mental health literacy; limited CaLD-appropriate mental health services; the perceived usefulness of ‘talking therapies’, and difficulty navigating Australia’s mental health system [29,30,31]. Conceptualisations of mental illness may have further implications for help seeking [18,30]. Arabic-speaking individuals who hold spiritual or religious beliefs about the causes of mental illness may be more likely to seek help from a religious leader [18] and mental health services may be seen as unnecessary for common mental illness, such as anxiety and depression [32].

### 1.3. Theoretical Framework

In order to develop a deeper understanding of the sources of help for mental illness, the current study was informed by the transactional model of cultural stress and coping [33,34]. Chun and colleagues [33] proposed a conceptual model of stress and coping, which comprehensively accounts for cultural context as well as transactions between person and environment. In this model, culture is viewed as an ecological system pervading the entire process of stress and coping, which comprises several panels: the environmental system, the personal system, transitory conditions, cognitive appraisal and coping skills, and health and wellbeing [33,34]. Chun and colleagues [33] stated that coping goals, in other words, the final outcome that an individual wants to achieve upon resolving a stressful situation, vary with culture, particularly across individualistic and collectivist communities. For example, individuals in a collectivistic community are more likely to set coping goals that embrace the needs of others as opposed to the needs of one’s self; reinforce relatedness and interdependence; control internal self, and minimise loss. Following on from coping goals, the coping strategies utilised are influenced by culture. It is hypothesised that collectivistic cultures focus on controlling and modifying one’s internal self (cognitive and avoidance-focused coping strategies) rather than strategies to confront external stressors.

### 1.4. The Current Study

To address the challenges that CaLD communities, predominantly from collectivist cultures, may experience when dealing with their mental health in Australia, a nation whose health systems and policies are primarily framed individualistically [35], a deeper understanding is required. In this paper, we explore the views of community members and leaders from the Arabic-, Mandarin-, and Swahili-speaking (Congolese) communities in Sydney, New South Wales. By exploring the commonalities and differences across these three groups regarding mental health sources of help, it is hoped that we will produce knowledge that can assist in the creation of the required policy directions for service provision.

## 2. Materials and Methods

### 2.1. Design, Study Context, and Population

This study was couched within a larger qualitative research project on mental-illness-related stigma in Arabic-, Mandarin-, and Swahili-speaking (Congolese) community leaders and members in Sydney, NSW. These communities were selected for the following reasons: Arabic and Mandarin are the two most spoken languages at home, other than English, and there is a growing intake of individuals from Congolese and Arabic backgrounds for humanitarian reasons. The study protocol was approved by the Western Sydney University Human Research Ethics Committee (H14608).

### 2.2. Participants’ Recruitment

Both purposive and snowball sampling was undertaken with the aim of obtaining homogenous samples and in accordance with Smith et al.’s [35] sampling strategy for interpretative phenomenological analysis (IPA). Participants were recruited using the networks of the relevant investigators and research staff. A $30.00 (AUD) electronic supermarket voucher was provided to all community members (focus group discussion participants) as reimbursement for their time in participating in the research.

The inclusion criterion for the key informant interviews was that the interviewee self-identified as a leader within the Arabic-speaking, Mandarin-speaking, or Swahili-speaking (Congolese) communities. This included both formal or informal roles, such as community worker, community elder or religious leaders. All were required to be older than or equal to 18 years of age and working in Sydney, Australia. The criteria for inclusion in the focus group discussions were that individuals had to be Arabic-speaking, Mandarin-speaking or Swahili-speaking (Congolese) with lived experience of mental illness, including the lived experiences of being carers or family members of those with mental illnesses. They had to be 18 years of age or older, residing in Sydney, and had to have arrived in Australia no more than seven years prior to the study. Due to specifics related to the Mandarin-speaking community, the length of arrival in Australia criterion was adjusted to include individuals who had arrived in Australia before the seven-year mark but due to continuous travel to China and back had not spent longer than a total of seven years residing in Australia. All focus groups were mixed-gender.

### 2.3. Data Collection

Eight focus group discussions and 26 key informant interviews were conducted during November and December, 2021. Six bilingual health workers from the target communities were employed and trained in the recruitment, screening, and interviewing process. Recruiting community members as bilingual health workers to assist in the data collection allowed building capacity within the community and enhanced the data collection process. To enhance rigour and data richness, this research project utilised a triangulated approach that incorporated data (by comparing community members and community leaders) and methodological triangulation (by incorporating both individual interviews and focus groups). The COREQ 32 [36] consolidated criteria for qualitative research were utilised and reported elsewhere [37].

In response to the 2021 COVID-19 Delta outbreak in Sydney and the quarantine restrictions, all interviews (focus groups and key informant) were undertaken remotely using Zoom and digitally recorded. Details on the process of screening, consenting and then undertaking both the focus group discussions and key informant interviews are detailed elsewhere [37].

Interview guides were generated following a literature review and input from the primary researchers [38]. Featuring a series of open-ended questions, the interview guides formed part of the larger qualitative research project. The questions focused on generating an understanding of each community’s conceptualisation of mental illness, including beliefs about aetiology and risk, barriers to seeking treatment, such as stigma, and sources of mental health help seeking.

### 2.4. Data Analysis

Interviews and focus group discussions were transcribed into written English by the bilingual health workers for the purposes of data analysis. This involved listening to the audio recordings and subsequently translating them from the original language to English, checking and re-checking the translations as required. A thematic analysis was conducted using the NVivo 12 software package and guided by interpretative phenomenological analysis (IPA) [35,38]. An IPA approach was used in order to obtain full and rich personal accounts and interpret the ‘lived experiences’ of the three CaLD community groups with respect to their views on sources of help for mental illness [39]. The quotes we present below are those we believe enhance our interpretation of the “lived experiences” of our participants.

Guided by IPA, the researchers attempted to make sense of how the participants make sense of their ‘lived experiences’, the ’double hermeneutic’ of this analytic process. In this process, IPA recognises that the thoughts, theoretical framework, and positioning of the researchers are central in their attempts to make sense of the subjective accounts of the participants. It is, thus, important to note that the theoretical positioning in the current study is one that views culture as an ecological system pervading the entire process of stress and coping, which comprises several panels [33].

Analysis was initially conducted separately by an author (psychologist) and experienced research officer and bilingual health worker. Weekly supervision meetings were undertaken with another author (psychologist) to resolve discrepancies and address issues of reflexivity. As noted in Slewa-Younan et al. [37], efforts were taken to address issues such as transcriptions and words. This process was not one-directional but bi-directional, as the members of the team who transcribed and those who were conducting the analysis were in communication. The initial codes were then grouped into categories. The next step involved searching for connections between categories, thus generating themes and subthemes. This process was then repeated for the next transcript. Once the analysis had been completed for each transcript, a final master list of themes was generated. At this stage, thematic analysis across the interview types and community groups was undertaken, generating the final results.

## 3. Results

Table 1 details the numbers and sociodemographic characteristics of participants. Most focus group discussion participants in the Arabic-speaking and Mandarin-speaking communities were female and aged 30 years or older. Conversely, participants in focus group discussions from the Swahili-speaking community were primarily male and aged 30 years or younger. All community leaders (key informant interviews) were aged 30 years or older.

Table 2 details a summary of the key themes and sub-themes.

**Theme 1: Informal Sources of Help for Mental Illness**
**Sub-theme: Social Sources of Help for Mental Illness**

All three communities highlighted that family, social, and community networks played an important role in managing mental illness. Seeking social support from trusted individuals or groups was often the first step in coping with mental illness.

“*A lot of people go to their closest friend*”

(Congolese FGD2, male, 21.)

“*It feels much better to talk to a friend.*”

(Mandarin FGD3, female, 40+.)

“*The Arabic community members have strong social ties, and they find a relieve from the daily stressors when they communicate with each other.*”

(Arabic interview, community leader, female, 71.)

Arabic-speaking participants noted that family members in particular play a significant role in supporting a person with a mental illness. Activities that encompassed family members and other forms of group support were most frequently identified as successfully managing mental illness, and the notion that changing the environment by taking the person to an outing was highlighted.

“*The closest to him should be around, take him out on a trip to change the atmosphere and adjust the psychological state, so he can forget his worries and the bad thoughts.*”

(Arabic FGD1, female, 61.)

Arabic-speaking leaders described how social events and gatherings with other people in the community can have a significant positive impact on the mental health of new arrivals.

“*We have seen many cases of individuals who have significantly improved just by socializing and integrating with the community*.”

(Arabic interview, community leader, male, 51.)

A contradiction was identified in the sense that while there was an awareness the person experiencing mental illness should not be isolated from the community and should be involved in activities, participants also stated that to avoid the stigmatisation and labelling of mental illness, mental illness was considered a private matter that should be managed within the family home. In this way, participants expressed attitudes both of paternalism and authoritarianism, as well as care and protection towards people experiencing mental illnesses.

“*The family hide the mentally ill person away and keep it as a secret*.”

(Arabic interview, community leader, male, 56.)

“*They try to hide him [individual experiencing mental illness] and deal with the issue without disturbing him or attracting his attention*.”

(Arabic FGD1, female, 61.)

In the Swahili-speaking community, the sources of help that were identified included friends, elders, and other leaders, who were seen as part of this collective network of support. For example, an elder from the community may be brought to a person to provide support and “counselling” and other community members may be asked to increase their support and care towards the person showing signs of mental illness.

“*When we elders especially find that someone has a change in their behaviour for example, when we ask from the community is to increase their care to the people. Sometimes if the person lives alone, we may find that the change in their behaviour is due to the isolation of the person in the community or in the family so we try to bring that person closer to the member of their family*.”

(Congolese interview, community worker, male, 57.)

Community groups were also identified as useful sources of help by leaders in the Swahili-speaking community.

“*So that’s why our Mother’s Group in our community now discusses mental health. In this group everyone discusses the state of their own mental health*.”

(Congolese interview, community leader, female, 41.)

“*This [The African Mental Health Learning Circle] is the platform where we go and actually try to break this stigma, this issue, into different points so that our people can actually grab the opportunity of getting to know what mental health is and how can it be treated, so that they can actually accept to receive the treatment that they need, for the betterment of their well-being*.”

(Congolese interview, community worker, male, 44.)

Within the Mandarin-speaking community, participants reported that individuals experiencing mental illness commonly receive social support from family and friends.

“*We didn’t know how to seek help from others and that family member who was deeply troubled was unwilling to seek help. Therefore, the main approach we took at that time was family companion, tolerance and understanding. At the same time, we actually encouraged him/her to try meditation and sports, which I think helped*.”

(Mandarin FGD1, female, 35+.)

Mandarin-speaking participants described WeChat as an effective platform to access social support from family and friends and promote community activities, such as painting and calligraphy workshops and sharing poetry. Such community-based coping strategies were identified as helpful because they facilitate a sense of social support and belonging for migrants who are separated from their social support in China. Participants mentioned that other vulnerable groups, such as seniors, were the target group pd workshops, such as handicraft workshops, singing, art groups, and dancing, ran by the Sydney Seniors Learning Society.

“*Participating in community activities every week, communicating with people online and talking about your difficulties are great ways to deal with mental health problems*.”

(Mandarin FGD3, female, 60+.)

**Sub-theme: Religious Sources of Help for Mental Illness**

Religious sources of help were discussed in all three communities but to various extents. In the Swahili-speaking Congolese community, besides seeking help from social sources, such as family and friends, seeking help from pastors was frequently mentioned. Moreover, it was clear from both the focus group participants and the community leaders that the lines between social sources of help, religious sources of help, and self-directed religious coping in the Swahili-speaking community were complex, as religiosity permeates the dominant model of care for those experiencing mental illness. Seeking help from pastors and faith-based coping was the primary way of coping with mental illness in the Democratic Republic of the Congo; this it appears remain the same after immigrating to Australia, especially among older community members. Church-based groups were also identified as sources of help in the Swahili-speaking community.

“*This is how the Congolese people address mental health, and that is not only back in Congo, even here in Australia, this is what is happening, we find out they go speak to the pastor*.”

(Congolese interview, community leader, male, 50.)

A religious leader stated that close and trustworthy relationships are formed with community members. In the context of a trustworthy relationship, individuals feel comfortable to discuss their problems.

“*Yes, to build that trust, I firstly became close friend to them. I have visited them sometimes by appointments. When I visited them, they cooked, and we ate together. In the Congolese culture, when you have such friendship, he/she will trust you and tell you everything. That’s trust and techniques. So, the first technique is to invite them, or they can invite me, and we will share a meal. At this level they will trust you and share with you, their problems. That’s I think religious leaders are trustworthy than others and psychologically it heals. For example, through talking stress can disappear without using any medication. Just talk to someone and they will be fine and that’s our secrets*.”

(Congolese interview, religious leader, male, 51.)

However, it was clear that not all participants viewed the role of pastors as important in managing mental illness in the Swahili-speaking community. Some noted that seeking assistance from pastors could prevent addressing issues that were likely to be contributing to development of the mental illness.

“*They may rely on a pastor instead of looking at what is causing them that stress*”

(Congolese interview, community health worker, female, 55.)

Others noted the impact that acculturation had on seeking assistance from pastors.

“*The young ones, who are brought up in Australia, have a different understanding of what is mental health. So [when] they are seeking support, they do go see few counsellors, and they do seek referrals from different organisations, but those who came from Africa, like their parents... seeking help to what I can easily tell you is very rare. They just go to the pastors, speak to them but seeing them to go see a counsellor or speak to a mental health practitioner, it’s very few that I’ve seen myself*.”

(Congolese interview, community leader, male, 50.)

Overall, it was clear that pastors held an influential and ‘gatekeeper’ role in the Swahili-speaking Congolese community, and this has direct service provision implications.

“*Church leaders are the most appropriate people to actually educate these people of the community for example, because in our community, people respect pastors so much, and if pastors say go to this particular situation, go and do this, people will run quickly to do that. So, it is the best way we do it*.”

(Congolese interview, community worker, male, 44.)

Among the Arabic-speaking participants, the leaders and focus group participants expressed differing views on the role of religion as a source of help when addressing mental illness. Notably, while self-directed religious coping in the form of prayers for the management of mental illness was discussed by Arabic-speaking community members, the direct input of a religious leader was not mentioned.

“*If a person is restless and anxious, as soon as he performs the prayers, his psyche relaxes and starting to feel better*”

(Arabic FGD2, female, 28.)

Conversely, leaders reported that seeking help from religious leaders, such as Imams, is common in the Arabic-speaking community because they are highly revered and trusted.

“*In my experience, the religion side plays a big role in this topic. People in general believe that the religious figure will keep the secret and will provide the right advice based on religion knowledge*.”

(Arabic interview, community leader, female, 59.)

A concern expressed by the Arabic-speaking leaders was the potential harm caused by those who mix religion and superstition in the Arabic-speaking community, and that these people take advantage of those seeking spiritual assistance.

“*We found an increase in unofficial traditional healers providing desperate people unrealistic solutions. There is a person I know is mixing the religion with superstitions, people should know the difference and seek help from medical professionals*.”

(Arabic interview, community leader, male, 56.)

While such unofficial traditional healers were not specifically mentioned in the focus groups, one participant alluding to the presence of such figures stated

“*In our Arabic countries, we believe more in superstitious treatment and magic more than we trust in medical treatment. I believe that ignorance is rampant within a group of people in the community, and they are not ashamed of it*”

(Arabic FGD2, Female, 41)

By contrast, religion played a lesser role within the Mandarin-speaking community. Instead, support within a church due to lack of other forms of social support when newly arrived was discussed. Such church groups were seen as providing members with a sense of belonging and support when experiencing mental health issues.

“*We helped her connect with a local church. Then, she went to the church to attend some activities, such as dancing, and a group of people accompanied her. I don’t know if it fits her, but I think if there’s such a problem, the church is definitely a good place to provide resources*.”

(Mandarin interview, community worker, female, 40+.)

**Sub-theme: Self-help as Source of Help for Mental Illness**

Participants in the Mandarin-speaking group discussed self-directed learning as a primary way of managing mental illness. This included engaging in hobbies and activities that can help increase feelings of competence as well as other strategies to regulate negative emotions.

“*Sports and hobbies will help*”

(Mandarin FGD3, female, 60+.)

“*Try not to think about annoying things*.”

(Mandarin FGD2, male, 60+.)

“*I’m more capable of adjusting myself, not to navel-gaze. I’d see things from a different angle, so it’s easier for me to get rid of bad mood*.”

(Mandarin FGD1, female, 50+.)

Engaging in self-directed learning of mental illness and the management of mental illness via seminars on YouTube, professional videos, attending psychology workshops, and reading articles was also mentioned.

“*I think there is no need to go to the psychologist because there are many seminars that target cases like me on YouTube. The seminars are quite comprehensive and pretty much cover everything. I personally find that what the psychologist said to me is as same as what I watched online. So, seeing a psychologist is just an opportunity for me to talk to someone. From my own perspective, I have known the problems already*.”

(Mandarin FGD2, male, 39.)

The role of self-reliance when dealing with mental illness was also highlighted both in the focus group discussions and interviews.

“*Mandarin-speaking people intend to put up with all things themselves. It is about endurance. For example, if you have some problems, you will regulate them yourself or deal with it yourself*.”

(Mandarin interview, community leader, female, 50+.)

Some focus group participants highlighted factors such as extensive wait-list times to see mental health professionals and wanting immediate and practical advice as contributing to self-directed learning as a preferred source help.

“*Psychologists also can help, but the psychologists would just listen to you. They cannot solve the problems at the end of the day*.”

(Mandarin FGD3, female, 60+.)

The Arabic-speaking community also referred to self-help to assist with mental illness, although this was undertaken in conjunction with the support of family or friends. For example, Arabic-speaking focus group participants regularly mentioned the benefits of changing one’s physical setting, particularly going “outdoors”, as well as increasing the uptake of activities, such as walking, to cope with mental illness.

“*All of us have depression or anxiety, we might suffer from it in the same day and we realise this, we take a walk, we go outdoor, do something to get rid of this feeling*”.

(Arabic FGD2, female, 47.)

Self-directed religious coping through prayer was also mentioned by the Arabic-speaking and Swahili-speaking focus group participants.

“*Prayer is where we go running to first with all our issues as we are religious*.”

(Congolese FGD2, male, 25.)

“*When it comes to recovery from illness it is not going to happen because of prayer alone we still need medical attention. Prayers will relax your soul and mental health only*.”

(Arabic FGD3, male, 35.)

**Theme 2: Formal Sources of Help for Mental Illness**

Formal sources of help for mental illness were discussed in all three communities, although to a lesser extent than informal sources. Within the Swahili-speaking Congolese focus group discussions, professionals such as general practitioners and counsellors were identified; however, the word psychologist was not mentioned. A few younger Swahili-speaking focus group discussion participants identified a counsellor as a more appropriate help-seeking option compared to self-directed religious coping via prayer.

“*If you are not feeling well mentally or your health you would go to your GP and you would tell them how you are feeling and after that the GP would refer you and if there is any counsellor. That counsellor would then tell you how you could go about your situation*.”

(Congolese FGD2, female, 27.)

In the Arabic- and Mandarin-speaking communities, sources of professional help were also discussed, especially if symptoms worsened and could no longer be managed with self-help activities or informal sources of help. Participants indicated that the decision to seek formal sources of help is initiated by the family.

“*If his health condition is advance, we must consult a doctor in this case, and it is not enough that you are the family dealing with him. In summary, at the beginning, it is the family and friends and if they cannot help, they need to seek professional advice*”

(Arabic FGD2, female, 28.)

Several leaders in the Arabic-speaking community stated that help is typically sought from general practitioners in the Arabic-speaking community, and a close relationship is usually formed between such professionals and community members.

“*The second group: people who settled in Australia for a few years (up to 10 years) and the main reason stopping these people from getting help from mental health professionals is they have built trust with their GP and they found it enough for them to vent for the GP*.”

(Arabic interview, mental health professional, male, 55.)

The effects of acculturation in facilitating more positive attitudes towards formal sources of help were mentioned by the Arabic-speaking leaders.

“*The second generation probably has a better understanding of how to reach professional help than the first generation and its more accepted to go and reach professional help on mental help than the first generation*”

(Arabic interview, religious leader, male, 43.)

Whilst most Mandarin-speaking focus group participants described a preference for informal sources of help, some focus group participants discussed their experiences of seeking professional help, primarily from psychologists, which were noted as positive by some and not helpful by others.

“*Well, I went to my GP first, and my GP referred me to a psychologist, who has helped me a lot. He/she has helped me improve a lot. I like my psychologist very much*.”

(Mandarin FGD1, female, 35.)

“*I have tried psychological counselling. Indeed, we are not confident about it. He/She would constantly listen to what you say, but he/she would not point out your thoughts or give any feedback on how to help you*.”

(Mandarin FGD1, female, 60+.)

## 4. Discussion

The findings of the current study identified the role and relative importance of informal and formal sources of help for mental illness in the Arabic-, Mandarin-, and Swahili-speaking communities. Below, we discuss the similarities and differences in the three communities’ perceptions of sources of help through the lens of the transactional model of cultural stress and coping.

Consistent with previous research [16,18,21], all three communities demonstrated a preference for seeking informal sources of help for mental illness compared to formal sources of help. Participants suggested that seeking social sources of help elicited feelings of belonging, connectedness, and alleviated distress, and this has been noted in previous research [40,41]. Similar to our finding, past studies have also demonstrated an association between collectivism and seeking social sources of help when experiencing psychological distress [33,42]. The environmental system of the model hypothesises that the coping support systems of collectivists encompasses multiple and larger social groups compared to individualists [33]. In order to leverage this, formal sources of help should be collaborating with informal sources of help to build these networks’ capacities and resources in order to better support individuals in their mental health and wellbeing. Moreover, formal service providers should thoroughly assess and be aware of the social supports available for coping when working with CaLD individuals (families). Keeping in mind that in most CaLD groups, working with an individual also encompasses their family.

The differences found regarding types of informal sources of help, both within and across the three communities, offer specific insights for service providers and policy makers alike. For example, Arabic-speaking participants noted that family members in particular play a significant role in supporting a person with mental illness. However, the centrality of the family in help-seeking decisions was not as strongly evident in the other two communities. Arabic-speaking participants also referred to self-help to assist with mental illness although this was undertaken in conjunction with the support of family or friends. Related to the value placed on interdependence, whether one’s family approves of professional help seeking is likely to impact the individual’s decision to seek help [21]. This strong influence of family on help seeking in the Arabic-speaking community has been consistently noted [43,44]. In line with the model’s predictions, our findings suggest that Arabic-speaking individuals may have a coping goal that reinforces relatedness and interdependence as opposed to autonomy and independence [33]. This coping goal also seems to be influenced by the need to ensure secrecy, and thereby maintaining family reputation by maintaining control over the individual with mental illness [21,45]. The individual experiencing mental illness may also view self-disclosure as a sign of weakness and betrayal to the family unit [44], particularly if a psychotherapeutic process demands disclosure of family secrets, and may threaten the sense of interdependence with others. Service providers should be sensitive to how these factors may be impacting CaLD individuals when they seek help within the mainstream health system. Arabic-speaking participants also alluded to regulating one’s psychological and internal state by going “outdoors” or engaging in social and community activities that enhance social relatedness and connection. In line with the value of collectivism, organising social and community events mobilises the community, as well as the capacity to heal within a collective [46]. Again, service providers and policy makers should take heed of social and community events being culturally appropriate environments that may be opportunities for effective entry points to promote help seeking.

Among the Congolese (Swahili-speaking) participants, a collective model of care for those experiencing mental illness was discussed. This model encompassed informal sources of help and was strongly influenced by religion. This collective and dominant model of care reinforces relatedness and interdependence in the group, and was seen as a way to offer healing relationships, in line with collectivistic coping strategies put forward by the transactional model [33]. Such coping can maintain group harmony and uphold hegemonic models of mental illness and coping. Religious leaders, specifically pastors, were portrayed by participants as ‘gatekeepers’ in the Congolese (Swahili-speaking) community. This view may have both positive and negative ramifications for help seeking. For example, black immigrant women in the USA identified spiritual resources as both sources of support and sources of stigma in the community [47]. Religious and community leaders are well placed to provide information to the community, which allows for well-informed mental-health-related decisions. Thus, our findings suggest that collaborating with community and religious leaders to enhance mental health education and literacy can assist in the development of culturally responsive care [48]. Indeed, harnessing the strong community support systems in Sub-Saharan African migrant communities can give rise to improved help seeking [16], with the African Mental Health Learning Circle, established 2016 being a notable example; it enables the exploration of mental-health-related issues and has partnered with both informal and formal help sources [49].

When considering notable differences, our Arabic discussion focus groups did not mention the role of seeking help from religious leaders, although the role of religious leaders in the Arabic community has been consistently noted in previous research [18,21,50]. While these differing findings may seem inconsistent, one must never disregard the heterogeneity that exists within specific CaLD communities, particularly with respect to religious practices and beliefs.

Unlike the other two communities, those in the Mandarin-speaking group discussed self-directed learning as a primary way of managing mental illness, the role of WeChat in supporting mental health and wellbeing, and community-based support for vulnerable groups, specifically seniors. The utilisation of self-help resources was identified in our findings and has been frequently reported in studies with East Asian migrants [32], specifically the practice of self-directed learning and the uptake of hobbies and activities to regulate internal emotional states. Although the self-reliant coping behaviour reported by the Mandarin-speaking participants may, on the surface, seem individualistic, contextualising this finding using the model speaks more to balancing the need for social harmony and the needs of the individual [33], reflected in the transaction between culture/transitory conditions and coping strategies. Mental illness may be viewed as an interdependent stressor because having a mental illness can have negative consequences on social and family reputation in Chinese communities [29]. In turn, Mandarin-speaking individuals may engage in self-reliant coping behaviour and also seek specific types of social support (i.e., coping strategies) to maintain privacy [42]. Mandarin-speaking participants, as opposed to Arabic-speaking and Swahili-speaking participants, also frequently discussed community-based coping activities, such as participating in painting and handicraft workshops. The values of interdependence and relatedness came through in the social sources of support identified by Mandarin-speaking participants. Seeking support from community-based groups, also viewed as intracultural coping, and social activities achieves a sense of belonging, protects group harmony, and minimises the likelihood of burdening others. That Mandarin-speaking individuals can participate in community-based groups to increase feelings of enjoyment, belonging, and competence while maintaining privacy provides clear insight into how the mental health of Mandarin-speaking migrants, who have been separated from traditional support structures, may be effectively supported. Furthermore, although such workshops may not directly target mental health, they offer a sense of social support and belonging in a culturally appropriate environment and represent an effective soft-entry point to formal help seeking.

A desire for practical and immediate advice from mental health professionals was also expressed by Mandarin-speaking participants. This preference for practical solutions has also been noted at an informal level. Na et al. [32] reported that Mandarin-speaking individuals were optimistic about resolving mental illness without seeking professional help. Relatedly, Chinese immigrants in Canada reported self-help strategies, such as physical activity, as being more helpful than other interventions, such as antidepressants [48]. Mental health professionals may be seen as ‘experts’ in the field, and correspondingly, there is an expectation that they are able to provide practical and immediate advice in line with cultural deference to authority. When such psychotherapeutic expectations are not met, this may reinforce individuals’ perceived low confidence in the helpfulness or competence of formal sources of help. A preference for self-directed learning should also be considered in light of practical barriers, such as difficulties navigating the mental health system in Australia by Chinese individuals. For Mandarin-speaking individuals, presenting programs to improve mental health as opportunities to learn, may be a more effective way of increasing uptake and accessibility.

All three communities mentioned formal sources of help for mental illness, although to a lesser extent than informal sources. In the Arabic-speaking and Mandarin-speaking communities, professional help sources were mentioned mostly when symptoms worsened. In the Arabic-speaking community, and consistent with prior research [21], participants indicated that the decision to seek formal help is typically initiated by the family. Comparatively, the Swahili-speaking community mentioned formal sources of help less frequently. Sub-Saharan African migrants may have alternative views on the appropriateness of formal sources of help, which may stem from historical and current experiences of racism and discrimination, as well as mistrust in the cultural responsiveness of Western mental health services [31,47]. Seeking help from Western mental health services carries the risk of misunderstanding and stigmatisation [31]. Indeed, certain mental disorders, such as schizophrenia, have been reported to be over-diagnosed in African American communities in the United States [30]. Moreover, the high levels of stigma of mental illness in the Congolese community and the desire to maintain group harmony, as well as protecting one’s needs may result in keeping mental illness a secret, which accounts for the limited mentions of formal sources of help. Service providers should be sensitive to how factors such as discrimination may be impacting individuals when seeking help within the mainstream health system. Our findings also suggested that differences in age and acculturation played a role in individuals’ the views of formal sources of help. For example, in the Swahili-speaking discussion focus group, participants who were younger in age had differing views regarding the dominant collective model of care. Our findings highlight the heterogeneity within and across communities and the importance of assessing the values, strengths, and resources of each individual.

We suggest that service providers should assess the coping goals of the individual and be aware that an individual may have several competing coping goals (i.e., balancing the needs of the individual and protecting group harmony), which may vary depending on factors such as acculturation and age. Moreover, access to health providers, such as general practitioners, who can speak the language of the person experiencing mental illness and is familiar with their cultural/religious background is likely to be an enabler of access to formal mental health care. Thus, policy advisors and education providers play a role in the development of such personnel, which, alongside working with informal sources of help, will help improve the mental healthcare of CaLD groups.

### Limitations and Strengths

Several limitations of this study should be noted. Firstly, the sociodemographic characteristics of participants, such as living in metropolitan Sydney, preclude the generalisability of the results to those living in regional or rural locations. Secondly, despite utilising a combination of purposive and snowball sampling, the possibility remains that individuals who are more active members in the community, such as those who attend Church groups, were recruited as opposed to members less active in community networks. As participation was voluntary, it may have resulted in the selection of a subset of individuals with more open attitudes toward mental health and illness. Furthermore, there is a possibility of social desirability bias, given that the key informant interviews were conducted with community and religious leaders who are often well regarded in their respective communities. Other limitations related more directly to methodology. Firstly, time and funding constraints precluded participants from the opportunity to review and check transcripts. Nonetheless, the interviewers did summarise the content of what was discussed at the end of each of the main sections of the interviews to ensure the participants’ perspectives were obtained. Notwithstanding these limitations, several strengths should be noted. Firstly, the research team and chief investigators were well integrated in their respective communities, allowing the development trust. Secondly, the interviews and focus group discussions were held in the language of each community, allowing the inclusion of newly arrived individuals who may have otherwise been excluded due to low English proficiency. Finally, as previously noted, training bilingual health workers allowed for capacity building within the CaLD groups, thereby improving their leadership and advocacy skills.

## 5. Conclusions

The importance of social sources of help was strongly noted across all three communities, with more nuanced roles held by religion and self-help activities. Formal sources of help were described by all communities, although to a lesser extent than informal sources. Our findings suggest that interventions to support help seeking for the three communities should involve building the capacity of informal sources of help. Notably, social support networks, which are culturally appropriate environments, can provide soft-entry points to foster collaboration between informal entities and individuals, such religious and community leaders, and formal sources, such professional mental health services. We have also highlighted differences between the three communities and have offered insights into the unique issues that need to be addressed for each community.

## Figures and Tables

**Table 1:** Sociodemographic characteristics of participants.

	Arabic-Speaking Community		Mandarin-Speaking Community		Swahili-Speaking (Congolese) Community	
	Focus Group Discussions	Key Informant Interviews	Focus Group Discussions	Key Informant Interviews	Focus Group Discussions	Key Informant Interviews
**Characteristics**	*n*	*n*	*n*	*n*	*n*	*n*
Gender						
Male	5	7	6	2	8	4
Female	13	3	15	6	4	4
Age Group						
18–30	3	-	1	-	9	-
30–39	4	-	6	2	2	-
40–49	7	2	3	3	-	4
≥50	4	8	11	3	1	4
Country of Origin						
Australia	-	1	-	-	-	-
China	-	-	21	8	-	-
Democratic Republic of Congo	-	-	-	-	12	6
Iraq	10	4	-	-	-	-
Other Middle East	-	4	-	-	-	-
Other Sub-Saharan Africa	-	-	-	-	-	2
Syria	8	1	-	-	-	-
Years in Australia since arrival						
≤7	18	-	17	-	12	-
>7	-	-	4	-	-	-

**Table 2:** Summary of key themes.

	Key Informant Interviews			Focus Group Discussions		
Themes	Arabic-Speaking	Mandarin-Speaking	Swahili-Speaking	Arabic-Speaking	Mandarin-Speaking	Swahili-Speaking
**Informal Sources of Help**						
Social Sources						
Family	+	+	+	+	+	+
Friends	+	+	+	+	+	+
Community member/s (individuals or groups)	+	+	+	+	+	+
Community leaders	+	+	+	-	-	+
Religious Sources						
Religious leaders (e.g., Pastor, Imam, Priest)	+	+	+	-	+	+
Church-based groups/activities	-	+	-	-	+	+
Unofficial traditional healer *	+	-	-	-	-	-
Self-Help						
Self-directed religious coping via prayer	+	-	+	+	+	+
Other self-help strategies	+	+	+	+	+	-
**Formal sources**						
General Practitioner/Doctor	+	+	+	+	+	+
Counsellor	-	+	+	-	-	+
Psychologist	+	+	+	+	+	-
Psychiatrist	+	+	+	+	-	-

Note. ‘+’ = Mentioned consistently in the group, or at least one member of the group; ‘-’ = Not mentioned in that group. * Unofficial traditional healer refers to individuals who mix religious and/or spiritual practices with traditional beliefs and are not part of larger official organisations.

## Data Availability

The data sets are not publicly available, as they contain information that could potentially re-identify individuals, but are available from SSY upon reasonable request and with relevant ethical approval.
